# Occupational Stress Among Health Worker in a National Dermatology Hospital in Vietnam, 2018

**DOI:** 10.3389/fpsyt.2019.00950

**Published:** 2020-01-24

**Authors:** Anh Nguyen Ngoc, Xuan Le Thi Thanh, Hue Le Thi, Anh Vu Tuan, Thanh Nguyen Van

**Affiliations:** ^1^Institute for Preventive Medicine and Public Health, Hanoi Medical University, Hanoi, Vietnam; ^2^General Examination Department, Quy Hoa National Dermatology Hospital, Binh Dinh, Vietnam

**Keywords:** occupational stress, JCQ-V, healthcare worker, hospital, Vietnam

## Abstract

A cross-sectional study was conducted among 171 doctors and nurses in a National Dermatology hospital using the Karasek's Job Content Questionnaire which has been validated in Vietnamese (JCQ-V), to assess the prevalence of occupational stress and to explore the association with some associated factors among them. The result showed that doctors and nurses with occupational stress accounted for 6.4%. This proportion was higher among nurse compared to doctor (8.0% vs. 2.2%); among those with diploma literacy compared to bachelor and above (10.6% and 2.3%). This rate was also higher in health workers under 30 years old (12.9%), health workers under 5 years at work (12.1%), working night shift from 3–4 nights (33.3%), temporary employment (12.8%), heavy workload occasionally (12.5%), and working hard occasionally (17.2%) compared to those in the comparison groups with p value <0,05. This prevalence concentrated in some departments such as surgery (11.9%), internal medicine (6.7%), dermatology, and others (1.5%). The study has not found the significant association between the prevalence of occupational stress and heavy workload and skill level. Therefore, it is essential for hospital should conduct screening all doctors, nurses, and medical staffs to identify subjects having occupational stress and give appropriate intervention.

## Introduction

Occupational stress is an imbalance between requirements and ability to work (1). In the context of globalization and changing factors of the nature of work, the environment is increasingly pressured and unstable, people are at risk of facing increasing work stress (2).

Burnout, a form of occupational stress was found to be common among junior doctors. In a recent global meta-analysis, more than half, 51.89% of dermatology residents suffered from burnout (3). Therefore, we carried out this project with the objective to identify the percentage of occupational stress using the Job Content Questionnaire in Vietnamese (JCQ-V) scale in doctors and clinical nurses at the national dermatology hospital.

In Vietnam, the issue of occupational stress among health workers has also been of great interest in recent years (4). Specifically, in 2016, a study conducted on eight central hospitals in Hanoi showed that 48.6% of health workers showed stress (5). A number of studies have focused on stress in clinical nurses and show that the incidence of occupational stress in this group was also relatively high (6–8).

In Vietnam, patients with skin diseases have low health related quality of life (9), low family quality of life (10), and poor sleep (11). More severe skin disease correlated to greater psychological burden (12) and such burden may be displaced to health professionals.

So far there has been no research on occupational stress among health staff in the Leprosy Hospital. Therefore, we carried out this project with the objective to identify the percentage of occupational stress using the JCQ-V scale in doctors and clinical nurses at Quy Hoa Central Leprosy-Dermatology Hospital in 2018 and to analyze some associated factors of occupational stress among health care work. The findings would help policy makers better understanding the situation and may develop appropriate intervention for reducing the occupational stress for good human resource in health facility in developing countries like Vietnam.

## Materials and Methods

### Research Subjects

Doctors and clinical nurses, with a labor contract of at least 1 year at the time of data collection and agree to participate in the study.

### Location and Study Time

The study was conducted at Quy Hoa Central Leprosy Dermatology Hospital from May 2018 to October 2018. The Hospital of Leprosy—Dermatology Central Quy Hoa is a class-1 hospital in dermatology that approved by Ministry of Health. The hazardous working condition of the hospital was ranked IV level by Vietnamese labor classification.

### Research Design

A cross-sectional research with quantitative method was applied in our study.

### Sample Size and Sample Selection

In our study, we found that 203 physicians and clinical nurses met the selection criteria. We interviewed 171/203 doctors and nurses working in the department of internal medicine, surgery, dermatology, and others (76.7%).

### Variable

In our study, we used dependent variable and independent variables. The dependent variable was occupational stress status (yes/no). The independent variables were some personal factors (included professional level, age, job age, gender, education level, …) and some elements of job characteristics (included department of work, average number of nights per week, workload, intense work, type of labor contract, …)

### Tools and Criteria for Assessing Stress

In our study, we used Karasek's model for assessing occupational stress. This model included 33 questions and assessed three aspects which were psychological stress (from 1 to 8), decision-making or self-control at work (from verses 9 to 25), and support through evaluating worker relationships with colleagues and superiors (from 26 to 33) (13). According to Karasek model, we classified stress into four group: high pressure jobs, passive work, active work, and comfortable work. High pressure jobs were calculated by total score psychological pressure over 16 and decision power ≤ 34 (threshold of occupational stress). Passive works were calculated by total score psychological pressure ≤ 16 and decision right ≤ 34. Active works were calculated by total score psychological pressure> 16 + decision right > 34. Comfortable works were calculated by total score psychological pressure ≤ 16 + decision right > 34. Stress status is “yes” when the assessment in the Karasek model is “high pressure work” (13). The questionnaire was tested on 02 subjects, including 01 doctor and 01 nurse, then completed before the official investigation.

### Data Processing

Data was entered, cleaned with Epidata 3.1 software and processing data using SPSS18 software. Test for the difference between the two proportions with the Chi square test or the Fisher exact test was used. Logistic regression was applied to measure association between occupational stress and some demographic characteristics. P value at 0.05 was significant level.

### Research Ethics

The study was completely approved by the leadership of Quy Hoa Leprosy Dermatology Hospital and was conducted after being approved by the council through the research protocol of the Institute of Preventive Medicine and Public Health, School. Ha Noi Medical University dated on April 2018. Collected information from people who agreed verbally to participate in research and information of research subjects was kept confidential.

## Results

### General Characteristics of the Object

We interviewed 171/203 doctors and nurses of hospitals, including 46 doctors and 125 nurses. Women accounted for a higher proportion than men (72.5% compared to 27.5%). Researchers group aged 30 and above accounted for 63.7%. More than half of research participants work for 5 years or more, at a rate of 61.4%. Half of research respondents have university and postgraduate educational level with the rate of 50.3% (**Table 1**).

**Table 1:** General characteristics of the sample group.

Variables	Characteristics	Frequency (n)	Rate (%)
**Age group**	< 30 years old	62	36.3
	≥ 30 years old	109	63.7
	$\bar{x}$ **± SD _(min–max)_: 34.64 ± 8.709_(23–65)_**		
**Gender**	Male	47	27.5
	Female	124	72.5
**Occupational age group**	1–5 years	66	38.6
	From 6 to 10 years	42	24.6
	Over 10 years	63	36.8
	$\bar{x}$**± SD _(min–max)_: 9.52 ± 6.757_(1–30)_**		
**Educational level**	Intermediate	85	49.7
	University Post graduated	86	50.3
**Specialist**	Doctor	46	26.9
	Nurse	125	73.1
Total		171	100

### Occupational Stress Status

Comment: According to Karasek model, the research participants with the most active jobs accounted for the highest proportion with 62%, followed by the group with comfortable work with 31.6%, the group feeling stressful work [love demand for psychology was high and decision-making power was low (6.4%)] and the group of research respondents who have to do passive jobs accounts for the lowest rate of 1.8% (**Table 2**).

**Table 2:** Occupational stress status of health workers accorsing to Karasek model.

Psychological stress	Decision right			
	*Low*		*High*	
***Low***	***Passive work***		***Comfortable work***	
	n	%	n	%
	3	1.8	54	31.6
***High***	***Stressful work***		***Active work***	
	n	%	n	%
	11	**6.4**	103	60.2

Bolded text denote for occupational stress among study participants.

The results in the **Table 3** show that there was a statistically significant relationship between stress rate and some personal factors (age group, education level), occupation factor (age group, ministry work division, number of nights, labor type, and intensity of work) with p < 0.05. Specifically, the rate of occupational stress in the research participants under 30 years old was 5.24 times higher than the participants over 30 years old (95% CI: 1.33–20.53, p value = 0.019 < 0.05). The rate of work-related stresses among researched junior and senior research participants was 4.97 times higher than that of university and post-graduate participants (95% CI: 1.04–23.75, p value = 0.036 < 0.05). The rate of occupational stress in the research participants under 5 years of age was 4.69 times higher than that in the participants working over 5 years (95% CI: 1.2–18.37, p value = 0.024 < 0.05). The rate of work-related stresses of surgeon staff was 8.8 times higher than that staff working in dermatology and other specialties (95% CI: 1.33–20.53, p value = 0.019 < 0.05). The rate of work stress of the research participants signing a contract was 5.1 times higher than that of the indefinite term (95% CI: 1.3–19.96, p value = 0.02 < 0.05). The rate of occupational stress in the research participants on duty three to four sessions a week was 12.4 times higher than that on the staff on duty two nights or less a week (95% CI: 3.3–46.7, p value = 0.00 < 0.05). The rate of occupational stress in the research group doing high intensity work with frequency (often or more) was 4.72 times higher than the group with the frequency of never or occasional (95% CI: 1, 34–16.7, p value = 0.02 < 0.05).

**Table 3:** Association between occupational stress and some characteristics.

Characteristics		Stress status				OR (95% CI)	p
		Yes		No			
		n	%	n	%		
**Specialist**	**Nurse**	10	8.0	115	92.0	3.91 (0.48–31.4)	0.292
	**Doctor**	1	2.2	45	97.8	1	
**Age group**	**< 30**	8	12.9	54	87.1	5.24 (1.33–20.53)	**0.019**
	**≥30**	3	2.8	106	97.2	1
**Occupational age group**	**< 5 years**	8	12.1	58	87.9	4.69 (1.2–18.37)	**0.024**
	**≥ 5 years**	3	2.9	102	97.1	1
**Educational level**	**Intermediate**	9	10.6	76	89.4	4.97 (1.04–23.75)	**0.036**
	**University** **Post graduated**	2	2.3	84	97.7	1
**Medical specialties**	**Surgeon**	7	11.9	52	88.1	8.8 (1.05–74.5)	**0.018**
	**Internal medicine**	3	6.7	42	93.3	4.71 (0.45–48.37)	0.15
	Dermatology and others*	1	1.5	66	98.5	1	1
**Number of nights on the week**	**3–4 nights per week**	6	33.3	12	66.7	14.8 (3.9–55.6)	0.00
	**under 2 nights/week**	5	3.3	148	96.7	1
**Type of labor**	**Limited contract**	8	12.7	55	87.3	5.1 (1.3–19.96)	**0.02**
	**Unlimited contract**	3	2.8	105	97.2	1	
**Large workload that exceeds the ability to work**	**Usually**	1	12.5	7	87.5	2.1 (0.2–19.5)	0.4
	**Never/sometimes**	10	6.1	153	93.9	1	
**Work with high intensity**	**Frequent/very often/Continuous**	5	17.2	24	82.8	4.72 (1.34–16.7)	**0.02**
	**Never/sometimes**	6	4.2	136	95.8	1	

Bolded and underlined texts denote for statistically significant.

In addition, the results in the table above also show that there was no statistically significant relationship between the occupational stress rate and some factors such as qualification and workload status (p value > 0.05).

## Discussion

### Occupational Stress Status

In this study, we used the JCQ-V scale to determine the occupational stress ratio of research respondents through three issues: psychological pressure, decision making power, and support from the working environment. This scale, developed by Karasek in 1998, has been assessed to be of good value and reliability and has been used in a number of previous studies in Vietnam. After investigating 171 subjects using a toolkit using the JCQ-V scale, based on Karasek's occupational stress model, we found that up to 62% of subjects were actively employed (pressure high psychological power, high decision-making power); 31.6% of the subjects were able to do a comfortable job (high decision-making power, low psychological pressure); 6.4% perceived to be high-pressure work (high psychological pressure and low decision-making power) and only 1.8% felt passive work (low psychological pressure, low decision-making power). This could be explained by the industry-specific nature, which requires a high degree of initiative, a high degree of decisive expertise in the professional activities of health workers, and less boring and simple work. More diversification so the work will also be more proactive and comfortable.

Thus, the results show that the occupational stress rate was 6.4%, which is similar to that of Pham et al. in 2011 in Hai Phong city using the JCQ-V scale (6.39%) (14). This rate is lower than that of Nguyen et al. in 2015 at Binh Dinh General Hospital (18%) (7), lower than that of Dang et al. in 2017 at Hanoi Medical University Hospital (6). This is lower than study in England (39%) (15), Portugal (11%) (16), Thailand (17,5%) (17), Taiwan (27%) (18), Germany (52%) (19), and India (63%) (20). This difference may be due to different research subjects. Our study selected all the doctors, clinical nurses in the hospital, the sample size was small (171), while the study of Nguyen et al. and Dang et al. selected only the subjects that were nurses and left across the rest of the subjects, large sample sizes (483). Due to the nature of work, nurses may be at higher risk of occupational stress than other subjects. Therefore, the assessment of stress in the population of only nurses would be higher than assessing the stress situation in the population including both doctors and nurses.

Another explanation is the difference in tools. The stress measurement tool of these two authors is DASS21. DASS21 is a validated scale to assess depression, anxiety, and stress in Asians (21, 22) and Vietnamese (23). The DASS21 tool uses seven questions, focusing on screening orientation to find cases of psychological stress in general, whether or not work-related. And the JCQ-V is a tool that explores the individual characteristics and description of the job (psychological pressure, decision making power, support in the workplace), using more questions (33 questions) to assessing workplace stress, an evaluation model that produces stress results will be tighter and may result in a lower rate. Therefore the difference in results is expected in advance. The different findings on the rate of occupational stress among studies would suggest that policy makers should develop a standardization tool to measure occupational stress among medical staff.

### The Relationship Between Stress Status According to Some Characteristics

In our study, we found that some characteristics related to stress status such as: age group and occupational age group; academic level; medical specialties; average number of nights per week; and labor contract. The number of people under 30 years old with work stress is 12.9%, while this figure in the remaining group is 2.8%, p = 0.019 < 0.05. This finding corresponds to findings of a recent meta-analysis which found that more than half, 51.89% of dermatology residents suffered from burnout (3). The occupational stress of young doctors and nurses could originate from depression, anxiety, and mental health issues encountered in medical and nursing schools (24–27).

The study has shown that the rate of occupational stress in the group with intermediate level and college is 4.97 times higher than in the research group with university and post university degrees (10.6% compared to 2, 3%, p = 0.036 < 0.05). The reason may be that the less educated people are, the more they are mentally pressured to become more professional, to improve their skills in order to keep up with the job, and to decide the job at work. They are lower than subjects with a university or higher degree, leading to a higher risk of stress.

Quy Hoa Leprosy Dermatology Hospital currently has 20 clinical departments divided into many divisions to manage, including: surgery, internal surgery, dermatology, and other specialties such as rehabilitation, traditional medicine, and department of examination. Each department in the hospital has different functions and tasks, specific characteristics of each department, so the assessment of the stress situation in each department is necessary to make appropriate judgments and interventions, suitable for medical staff in the department. Through univariate analysis, we found that, compared to the group working in the dermatology group, those working in the surgeon sector suffered from occupational stress with the rate of 8.8 times higher (11.9% compared to 1.5%). This result similar to research in China in 2017 and India in 2018 (20, 28). This is consistent with the reality at the hospital. The surgeon division is a new specialized division invested in recent years, which is being developed in the hospital. Due to the characteristics of the surgeon division, most of the employees have to work with sick people who are quick to respond, highly responsible, time is not proactive, pressure from family members, often in contact with their lips. The risk of infection is caused by exposure to bodily fluids, which makes the occupational stress higher. While those who work in the dermatology division, there are fewer endangered diseases and this is also a specialized branch of the hospital so the highly qualified human resources will be less stressed at work. Because no studies classify this as ours, comparisons for this result are not possible.

Night duty is a specific task for health workers and has a significant impact on health due to changes in the normal circadian rhythm of humans. The majority of shift work affects sleep after the night shift and the quality of sleep before the morning shift. The combination of sleep deprivation and working at a time when bodily functions are restricted can cause severe fatigue and insomnia, difficulty in performing good jobs and increase the risk of accidents, and stress (29). The study results showed that, occupational stress in the on-call group for 3–4 nights/was 33.3%, 14.8 times higher than the lower group less than or equal to two sessions a week (33.3% vs 3.3%, p = 0.000 < 0.05). This result is similar to the result of Nguyen Van Tuyen on nursing at Binh Dinh General Hospital in 2015, those who are on duty more than two nights a week are 1.9 times more likely to stress than those who only directly from two nights/week or less with p = 0.02 (7). Another study at the Hanoi Oncology Hospital also showed that clinicians who were on duty four times a month were 6.8 times more likely to be stressed than those under four sessions per month (8).

Our results show that those with a fixed term contract had a Gaaos stress rate of 5.1 times that of an indefinite or permanent contract (12.7% compared to 2.8% p = 0.02 < 0.05). Perhaps with the mentality of wanting a stable career, those who sign indefinite contracts—usually young people—are just out of school, are in the process of demonstrating competence before being put on an indefinite preparations. Therefore, they will have more psychological effort and higher pressure to show their position at work, leading to a higher risk of occupational stress than the other group.

Because of time and manpower limitations, our study was conducted on only doctors and nurses in a hospital, so it was not possible to represent the entire healthcare workers. This is a cross-sectional descriptive study that investigates the status and factors that affect the stress of medical staff, so it is not possible to confirm what is the main cause of the stress. This suggests more in-depth, complete, and multi-dimensional studies are needed to make clearer and more accurate judgments.

## Conclusions

Findings indicated that the research group with the most active jobs accounted for the highest proportion (62%). The job stressors affecting the healthcare workers included some personal factors (age group, education level), occupation factor (age group, ministry work division, number of nights, labor type, and intensity of work). Hospital should conduct screening all doctors, nurses, and medical staffs to identify subjects having occupational stress and give appropriate intervention. For doctors and clinical nurses, the hospital needs to enhance the exchange of sharing experiences, sharing the work volume. Next studies should use a standardized tool to assess occupational stress and follow-up data to evaluate the effectiveness of interventions.

## Data Availability Statement

The datasets generated for this study are available on request to the corresponding author.

## Ethics Statement

The studies involving human participants were reviewed and approved by Hanoi Medical University Committee dated in April 2018. The patients/participants provided their written informed consent to participate in this study.

## Author Contributions

Conceptualization: XL, AN, HL, AV, and TN. Data curration: AN, and XL. Formal analysis: AN, and XL. Investigation: HL, and AV. Methodology: AN, and XL. Project administration and supervision: XL, AN, and AV. Visualization: AN, and XL. Writing-original draft: AN, XL, TN, HL, and AV. Writing review and editing: AN, and XL.

## Conflict of Interest

The authors declare that the research was conducted in the absence of any commercial or financial relationships that could be construed as a potential conflict of interest.
